# Model-Integrated Bioequivalence (MIBE) in Generic Drug Research: Can We Ease the Bioequivalence Burden?

**DOI:** 10.3390/pharmaceutics18050536

**Published:** 2026-04-28

**Authors:** Sivacharan Kollipara, Rajkumar Boddu, Chandra Teja Uppuluri, Anuj Kumar Saini

**Affiliations:** Biopharmaceutics Group, Global Clinical Management, Dr. Reddy’s Laboratories Ltd., Integrated Product Development Organization (IPDO), Bachupally, Medchal Malkajgiri District, Hyderabad 500090, Telangana, India; rajkumarboddu@drreddys.com (R.B.); uppulurichandrateja@drreddys.com (C.T.U.); anujkumarsaini@drreddys.com (A.K.S.)

**Keywords:** MIBE, PBPK, PBBM, regulatory, bioequivalence, alternative BE, generic product development

## Abstract

Bioequivalence (BE) studies are essential to file an abbreviated new drug application (ANDA) against an innovator drug product. Conventional BE studies can be complex, time-consuming, and operationally challenging, particularly for products with long half-life drugs, high variability, or formulation complexity. Advances in quantitative modeling and simulation have expanded the role of model-generated information in generic drug development from a supportive role toward providing critical regulatory evidence. Model-Integrated Bioequivalence (MIBE) represents a focused application of this paradigm in which mechanistic or empirical models are used to directly support BE determination. While physiologically based pharmacokinetic (PBPK) and physiologically based biopharmaceutics modeling (PBBM) approaches have been widely discussed in the literature, increasing attention is being directed toward population pharmacokinetic (POP-PK) modeling for MIBE implementation, particularly when mechanistic assumptions are uncertain or extensive in vitro characterization is impractical. This review provides a contemporary overview of MIBE in generic drug development, with a specific emphasis on POP-PK-based approaches. Key quantitative modeling frameworks are discussed along with evolving regulatory perspectives that support the integration of model-based evidence for BE assessment. We illustrate six diverse hypothetical case examples covering different formulations, a variety of BE scenarios and using MIBE to answer specific regulatory questions on BE. Collectively, this manuscript addresses an important topic of MIBE for complex and non-complex generic formulations and may provoke thinking among the generic companies to use such approaches in the regulatory context to enable faster and timely approval to bring the necessary medicines to the market at a rapid pace.

## 1. Introduction

For generic drug products, the demonstration of bioequivalence (BE) with a reference listed drug (RLD) is a critical regulatory requirement for product approval. As defined in 21 CFR 314.3 (b), BE is “the absence of a significant difference in the rate and extent to which the active ingredient or active moiety in pharmaceutical equivalents or pharmaceutical alternatives becomes available at the site of drug action when administered at the same molar dose under similar conditions in an appropriately designed study” [1]. In practice, BE is most commonly demonstrated through comparative pharmacokinetic (PK) studies in healthy volunteers.

Conventional in vivo BE studies are typically conducted using crossover, replicate crossover, or parallel designs depending on the PK characteristics of the drug substance and formulation design. Plasma concentration–time profiles based on an appropriate sampling scheme are obtained and key PK parameters are computed for test and RLD treatments. Bioequivalence is concluded when the 90% confidence intervals (CIs) for the geometric mean test-to-reference (T/R) ratios of key PK parameters—most commonly maximum plasma concentration (C_max_) and area under the concentration–time curve (AUC)—fall within the regulatory acceptance range of 80.00–125.00% [2,3,4]. While this is the default framework for a large number of immediate-release and simple formulations, it can become increasingly burdensome or impractical for products with complex PKs, long half-lives, high variability, or formulation-specific challenges.

Beyond the PK-based BE studies, evidence of BE can also be obtained from in vitro, pharmacodynamic or comparative clinical end-point studies, depending on the drug and drug product characteristics, site of action, etc. For example, for the product-specific guidance (PSG) for mesalamine extended-release oral capsules, ref. [5] mentions the requirements for bioequivalence studies as well as demonstration of in vitro dissolution similarity between test and reference formulations. The product-specific guidance for Tapinarof cream [6] provides two options—(i) two in vitro BE studies or (ii) one in vivo comparative clinical endpoint study based on the composition similarity between the test and reference product. In case of oral extended-release formulations such as Topiramate [7], apart from conventional fasting and fed bioequivalence studies, a fasting sprinkle bioequivalence study is also required to demonstrate BE with RLD. These examples highlight that BE requirements are highly product-specific and are tailored to formulation complexity and risk.

In recent years, the role of modeling and simulations (M&Ss), quantitative modeling methods (QMMs), and model-informed drug development (MIDD) has expanded substantially in generic product development. Although such approaches have long been integral to new drug development, their systematic adoption in the generic industry has gained momentum more recently. Applications of these approaches in the generic setting have been reviewed extensively in the literature, with biopharmaceutics identified as a key area of impact [8,9].

Within the biopharmaceutics domain, physiologically based pharmacokinetic (PBPK) and physiologically based biopharmaceutics modeling (PBBM) tools have evolved substantially. These mechanistic models integrate drug physicochemical properties (like pH–solubility profiles, solubility data in biorelevant media, pKa, log P, etc.), formulation attributes, systemic PK parameters, and physiology to simulate in vivo drug performance under a wide range of clinical scenarios. PBPK and PBBM models have been applied to evaluate formulation changes, establish in vitro–in vivo correlations or relationships (IVIVC/IVIVR), support scale-up and post-approval changes, justify lower-strength biowaivers, address dissolution (f2) mismatches, and enable virtual assessments of food effects, drug–drug interactions, or altered physiological conditions [10,11,12,13].

Complementing these mechanistic approaches, quantitative clinical pharmacology (QCP) models are being increasingly applied to support risk-based BE strategies and narrow therapeutic index (NTI) drug assessments. A particularly important application of QCP modeling is in the development of long-acting injectable (LAI) products, where model-based analyses are used to identify partial AUC (pAUC) metrics and inform BE recommendations for products with complex, extended-release profiles [8,9,14,15].

Population pharmacokinetic (POP-PK) modeling represents another quantitative framework that has followed a similar trajectory—well established in new drug development through MIDD principles and more recently adopted within generic drug development. This empirical or semi-empirical tool has shown particular utility in the development of LAIs due to potential applications in reducing the study duration, shortening the washout period, handling missing samples, etc. [16,17,18]. Beyond LAIs, Pop-PK modeling is increasingly being deployed in situations where development of fully mechanistic PBPK or PBBM models may not be feasible or necessary [19,20].

Historically, modeling approaches in generic settings were applied in a supportive capacity—for formulation selection/optimization, guiding study design, extrapolating in vivo BE findings to alternative clinical or physiological scenarios. More recently, we are witnessing a paradigm shift toward the use of model-generated information as critical evidence in regulatory decision making. In a recent workshop presentation, the FDA defined model-integrated evidence (MIE) as “*using model generated information such as the virtual bioequivalence (VBE) study results not just to plan a pivotal study but to serve as pivotal evidence*” [21,22].

Model-Integrated Bioequivalence (MIBE), a focused application of MIE to BE determination, has gained momentum within the FDA’s generic drug program, formalized through the FDA MIE Industry Meeting Pilot Program launched in October 2023. Under this framework, multiple sources of information—including in vitro data, clinical PK data, and mechanistic or empirical models such as PBPK, PBBM, Pop-PK, QCP, or their combinations—are formally integrated to enable virtual or partially virtual BE assessments [21,22]. This evolving MIE paradigm reflects growing regulatory openness to replacing or reducing conventional in vivo BE studies. As model-derived information increasingly contributes to regulatory decision making, commensurate emphasis is placed on establishing model credibility through clearly defined contexts of use, rigorous model development and evaluation, and explicit consideration of decision risk, including control of type I errors and statistical power [21].

In this context, the objective of the present review is to provide a contemporary overview of model-integrated bioequivalence in generic drug development, with a specific emphasis on population pharmacokinetic approaches. While PBPK and PBBM methodologies have been extensively reviewed elsewhere, this manuscript focuses on the application of MIBE principles using Pop-PK models, including discussion of development workflows, validation considerations, and regulatory contexts.

The novelty of this review lies in the presentation of illustrative, hypothetical case studies that demonstrate diverse applications of MIBE across formulation types and bioequivalence scenarios. Through these examples, the manuscript aims to provide readers with a practical, conceptually grounded understanding of quantitative modeling approaches in generic drug development and their potential role in enabling efficient, science-based regulatory decision making.

## 2. Model-Integrated Bioequivalence (MIBE)

MIBE can broadly be defined as a regulatory approach using modeling and simulations in place of traditional clinical trials to provide pivotal evidence that the generic product is bioequivalent to the RLD. This methodology enhances the efficiency of generic drug approval especially for complex drug products, thereby reducing the need for multiple, costly, lengthy in vivo studies and supporting abbreviated new drug applications (ANDAs).

The MIBE framework may encompass any type of model, including PBPK, PBBM, POP-PK or QCP models, depending on the specific cases and context of use being considered. MIBE is typically applied to complex drug products having scarcity of data or exhibiting lack of in vitro–in vivo correlation. For example, in the case of long-acting injectables (LAIs) or inhaled or topical products, it may not be feasible to have multiple pilot studies to validate the models and to establish in vitro dissolution (or any other characteristic) that is representative of in vivo behavior. In such cases, empirical models such as POP-PK can be developed for the purpose of MIBE. Similarities and differences among POP-PK, PBPK/PBBM are presented below in Table 1 [21,23,24].

As outlined above, MIBE—particularly when implemented using population pharmacokinetic models—is an evolving regulatory paradigm with multiple potential applications in generic drug development. Pop-PK-based MIBE approaches have been explored to address challenges such as waiving or simplifying pivotal BE studies, shortening study durations for long-acting products, correcting for carryover effects in crossover designs with long washout requirements, optimizing study design and sampling strategies, and accounting for inter-individual variability in pharmacokinetic exposure [16,18]. These applications are especially relevant for long-acting injectables (LAIs), where formulation complexity, extended sampling windows, high dropout rates, and operational constraints frequently complicate conventional BE assessments.

Development of an MIBE strategy begins with clearly defining the regulatory context of use and the specific question of interest (QOI), including key details like the product characteristics, route of administration, dosing conditions, variability considerations, and whether the intended application involves study waiver, extrapolation, or study modification [22]. This is followed by selection of an appropriate modeling framework—PBPK/PBBM, Pop-PK, or a hybrid approach—commensurate with data availability and the desired context of use.

A transparent and prespecified model development strategy is critical to MIBE implementation. This includes clear documentation of data sources, model structure, parameter estimation methods, diagnostic criteria, and validation approaches. In the case of POP-PK models, evaluation typically involves assessment of goodness-of-fit diagnostics, residual distributions, visual predictive checks, and sensitivity to uncertainty assumptions, which need to be clearly documented and pre-specified.

When a model is proposed to provide critical evidence for BE determination, explicit consideration of decision risk becomes essential. Controlling type I errors (consumer risk, i.e., the probability of wrongly inferring a non-bioequivalent drug product as bioequivalent) at the regulatory threshold of not more than 5% and assessment of statistical power (manufacturer’s risk) are integral components of POP-PK-based MIBE evaluations.

Once validated and shown to be fit for purpose, the POP-PK model may be applied to address the predefined regulatory question—such as waiving a BE study, extrapolating results from a smaller dataset (pilot) to a larger population (pivotal), mitigating carryover effects, or reconstructing full PK profiles from sparse sampling designs. Model credibility, including its impact on decision making and the consequence of potential error, must be evaluated holistically prior to regulatory application [25].

**Figure 1:**
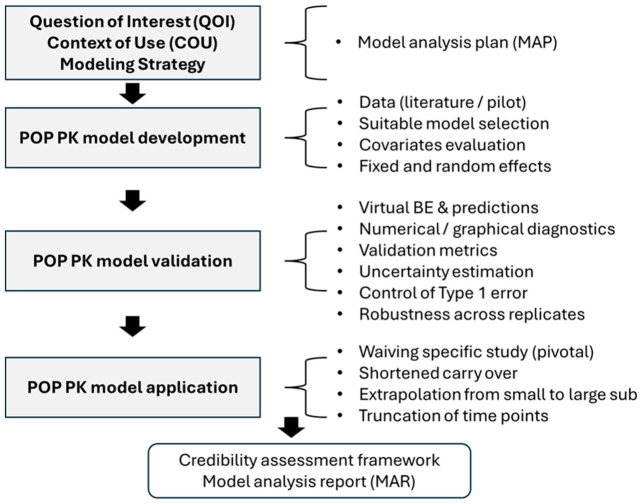
MIBE workflow for the POP-PK models.

A generalized workflow illustrating key steps involved in implementing POP-PK-based MIBE is summarized in Figure 1.

Practical application of POP-PK-based MIBE in a regulatory context has been illustrated in a limited number of published examples. One such case was reported by Gopalakrishnan et al., where a POP-PK-based MIBE approach enabled avoidance of potential repeat pivotal studies for clopidogrel bisulfate 300 mg tablets [26]. In that instance, the original pivotal study employed a shorter sampling window, resulting in a request for a repeat study with extended sampling. As a response, POP-PK models were developed using pilot study data in both fasting and fed conditions and subsequently validated against pivotal study data. Control of type 1 errors in the model was demonstrated successfully. The validated model was then applied to extrapolate exposure metrics to the agency-requested sampling duration, demonstrating that the original sampling scheme did not compromise BE conclusions, thereby obviating the need for a repeat BE study [26].

Recently, the concept of MIBE has been a topic of discussion in the scientific domain. Nyberg et al. evaluated MIE strategies for PK BE studies using model averaging methods [27]. Two model averaging methods were examined: bootstrap model selection and weight-based model averaging with parameter uncertainty from three different sources, either from a sandwich covariance matrix, a bootstrap, or from sampling importance resampling (SIR). These approaches were found to have superiority over traditional NCA-based methods [27]. The research from Bois et al. indicated the use of a Bayesian framework for virtual comparative trials and BE assessments [28]. The Bayesian framework is used to determine the safe space and was found to be more precise than frequentist workflows [28]. The research performed by Mollenhoff et al. highlighted the use of efficiency in model-based bioequivalence testing [29]. It was discussed that NCA is not a reliable approach in the case of sparse designs, and a model-based alternative approach was proposed for estimation of AUC and C_max_ using NLME models. This method appears to control type 1 errors at the applicable level of 0.05 and thus can be used in situations where conventional BE is not applicable. Further, alternative approaches have been provided in this manuscript [29]. In another paper, Hsieh et al. indicated the use of Bayesian population modeling and virtual bioequivalence assessment approaches to establishing dissolution specifications for oral dosage forms [19]. These studies clearly indicate the progress in MIBE and its potential as an alternative compared to NCA for efficient BE assessments for complex drug products.

Taken together, these examples and methodological developments illustrate that the scientific basis for Pop-PK-based MIBE is increasingly well established. These advances position population pharmacokinetics as a central quantitative tool for addressing complex BE challenges and provide a strong foundation for the evolving regulatory perspectives discussed in the following section.

## 3. Regulatory Perspectives of MIBE

In parallel with advances in modeling methodologies, regulatory agencies have increasingly articulated pathways for the integration of model-based evidence into generic drug development and assessment. The United States Food and Drug Administration (USFDA) has taken a leading role in formalizing this with the launch of an MIE pilot program in the year 2023 [21]. This program enhances the communication between industry and the regulatory body on the use of quantitative modeling techniques to improve efficiency, scientific rigor, and predictability in generic product evaluation. A few examples of MIE include model-based bridging between two dosage forms, the use of mechanistic PBPK modeling to use fewer subjects and extrapolate outcomes to larger populations for reducing the recruiting burden for patients [21,30,31,32].

Regulatory perspectives on MIBE have also been shaped through a series of FDA-led and collaborative scientific workshops focused on modeling applications in generic drug development.

The 2017 workshop on “Model-Informed Drug Development and Review for Generic Products: Summary of FDA Public Workshop” deliberated about the importance of modeling tools in generic product development [24]. The speakers talked about use of POP-PK approaches in optimal study design and comparison of NCS vs. POP-PK approaches. This publication from the USFDA highlighted generating model-integrated evidence for generic drug product development and the use of QMM in BE assessment for locally acting drug products and complex and modified-release drug products. Further, the use of QMM for complex products such as mesalamine, methylphenedate, abluterol inhalation products, levonorgestrel, ivermectin topical creams, and oxybutynin in addressing regulatory questions was portrayed with practical insights. The FY2021 workshop on Generic Drug Regulatory Science Initiatives has further provided insights on data analysis and model-based bioequivalence for generic drug products [16]. In this workshop, the speakers portrayed the use of pharmacometrics models to understand and optimize study designs, to support switch studies for LAI formulations, and focused on the concerns of steady-state calculations with conventional methods. Additional focus was made on model and systems-based approaches to address efficacy and safety questions related to generic submissions [16].

The workshop “Model-Informed Drug Development for Long-Acting Injectable Products: Summary of American College of Clinical Pharmacology Symposium” extensively focused on the use of MIBE to tackle BE challenges of LAIs, namely longer sampling duration, higher dropouts, steady-state studies in patients, significantly higher washout in case of crossover designs, etc. [18]. Further, efficient BE analysis was discussed for LAIs that include model-informed and model-integrated approaches to support LAI BE studies. Further, the workshop on “Establishing the Suitability of Model-Integrated Evidence (MIE) to Demonstrate Bioequivalence for Long-Acting Injectable and Implantable (LAI) Drug Products” [32] portrayed in vitro, in vivo BE and use of MIE approaches in generic drug development. This conference focused on MIBE for LAIs in conducting the complex BE studies and ways to optimize them. Further, it also highlighted GDUFA-sponsored research in MIDD and grants available in this area. Challenges when using PK endpoints such as sparse PK sampling, endogenous baseline corrections, and long study durations for LAIs that can pose challenges in practice and assessment were discussed in detail [32].

Collectively, these regulatory initiatives and scientific exchanges indicate that MIBE is transitioning from an exploratory concept toward a regulated, structured approach within generic drug development. Population pharmacokinetic modeling, in particular, has been consistently emphasized as a practical and scientifically robust tool for enabling MIBE in settings where mechanistic modeling may be infeasible or conventional BE studies are disproportionately burdensome.

### Considerations, Guardrails and Limitations for Regulatory Use of MIBE

MIBE offers a powerful, science-based alternative to conventional BE studies when applied within a clearly defined regulatory context of use (COU). Regulatory acceptance of model-integrated evidence is inherently risk based and depends on the quality, relevance, and representativeness of data used for model development. Models intended to support pivotal BE decisions must therefore be grounded in fit-for-purpose clinical, in vitro, and prior knowledge, with transparent articulation of assumptions and their potential impact on predicted outcomes.

From an industry perspective, this may introduce operational challenges that in-crease upfront burden/complexity over conventional approaches. For example, model-based approaches may need to demonstrate model discriminatory power by deliberately evaluating non BE or borderline formulations. Additional pilot studies, exploratory experiments, or sensitivity analyses may be required as a part of verification and validation (V&V) activities. Early and iterative engagement with regulatory agencies at stages is another key difference that is traditionally unconventional for generic development. Such activities demand significant internal alignment across formulation, clinical, pharmacometrics, and regulatory functions.

Although these requirements introduce upfront complexity and operational friction, they are essential to establish confidence in model-integrated evidence. As discussed above, these efforts can ultimately enable regulatory acceptance of MIBE and meaningfully reduce down-stream BE study burden without compromising decision integrity.

Further, to provide practical perspectives on the MIBE approaches, we portrayed six different and diverse hypothetical case examples covering various types of formulations and modeling approaches to provide an overview of the potential of such approaches in generic product development. Along with the case examples, we also portrayed their benefits as alternative BE approaches against the conventional tools in terms of cost and time savings to enable early generic product launches in the market.

## 4. Case Examples

To illustrate the practical application of Model-Integrated Bioequivalence (MIBE) across diverse product types and regulatory questions, a series of case examples are presented in this section. These cases span a range of formulations, pharmacokinetic behaviors, and development challenges, and collectively demonstrate how different quantitative modeling approaches—including nonparametric methods, POP-PK models, and physiologically based biopharmaceutics modeling (PBBM), etc.—may be leveraged within an MIBE framework.

The primary intent of these hypothetical case examples is illustrative. They are presented as proof-of-concept demonstrations of how MIE can be structured to address specific BE questions, rather than as fully developed regulatory submissions.

The examples rely on dummy pilot-scale or single-study datasets, assume linear PK for the drugs, and employ modeling approaches whose verification and validation are limited to within-study diagnostics or presumed literature support. Comprehensive external validation, extensive sensitivity analyses, and full operating-characteristic evaluations (e.g., systematic type I error and power assessments across virtual trials) were not performed for all cases. Accordingly, these examples should be interpreted as conceptual demonstrations of MIBE feasibility rather than prescriptive regulatory templates.

Each case illustrates a distinct regulatory question and context-of-use for MIBE, with the selected modeling approach aligned to the nature of the formulation, data availability, and decision consequence. A summary of the six case examples discussed in subsequent sections is presented in Table 2.

### 4.1. Case Study 1: Applicability of MIBE for Waiver of Multi-Dose Bioequivalence Study

#### 4.1.1. Objective

To evaluate whether an MIBE strategy based on empirical nonparametric superposition of single-dose PK data could credibly predict steady-state exposure and bioequivalence (BE) for an ER product, thereby supporting a waiver of the clinical multiple-dose BE requirement in the EU setting.

#### 4.1.2. Context and Rationale

For modified-release products, some regulators require BE to be demonstrated at steady state unless accumulation after a single dose is minimal.

In the present case, single-dose BE between the test and reference ER formulations was established under fasting conditions (n = 27), but the accumulation fraction derived from single-dose data was ~85% for both products—below the 90% threshold that can justify waiving a steady-state study under EMA expectations. Hence, a clinical multiple-dose study at the same sample size (n = 27) was therefore conducted and showed BE at steady state.

This case study explores whether a prospectively applied MIBE approach—using nonparametric superposition of the observed single-dose profiles—could have predicted the steady-state BE outcome to support a waiver, thereby reduce the in vivo BE study burden.

#### 4.1.3. Methods (MIBE Workflow)

***Tools and software***: A nonparametric superposition-based MIBE approach was implemented in Phoenix WinNonlin (version 8.6) to simulate steady-state profiles from observed single-dose plasma concentration–time data; the same platform was used for noncompartmental analyses and BE statistics.

***Study data and design***: The data from observed single-dose and multiple-dose BE studies was employed. A randomized, two-treatment, two-period, single-dose crossover (fasting, n = 27) that established BE for C_max_, AUC_0–t_, and AUC_0–∞_. Accumulation estimated from single-dose data was approximately 85% (test 85.37%; reference 84.64%), triggering the need for a clinical steady-state assessment per EU expectations. A multiple-dose (n = 27) study was then performed, confirming BE at steady state for C_trough_, C_max,ss_, and AUC_τ_.

***Simulation and analysis***: Using the observed single-dose profiles for test and reference for each subject, nonparametric superposition was applied to simulate repeated once-daily dosing through six doses (reached steady state). Simulated steady-state profiles at dose six were summarized via NCA to derive C_max,ss_, AUC_τ_, and C_trough_. Virtual BE comparisons were generated from the simulated data and compared against the observed steady-state BE study outcomes. Concordance was assessed using prediction errors for key parameters.

#### 4.1.4. Results and Conclusions

The single-dose study confirmed BE in the fasting state with respect to C_max_, _AUC0–24_, AUC_0–tlast_, and AUC_0–∞_ (Table 3). The multiple-dose clinical BE at the sixth dose showed that test and reference remained bioequivalent at steady state for C_max,ss_, AUCτ, and Ctrough.

**Figure 2:**
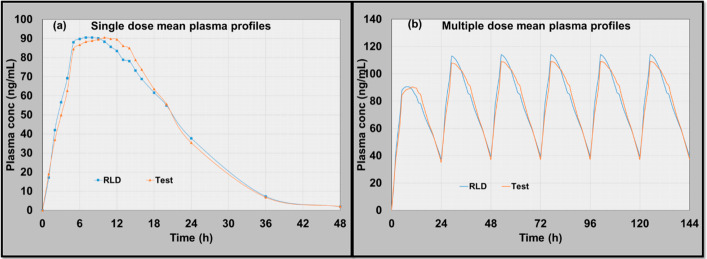
(**a**) Observed mean single-dose PK profiles and (**b**) mean of simulated steady-state PK profiles of reference and test products.

Critically, the simulated steady-state BE results derived from nonparametric superposition closely matched the observed steady-state outcomes. Across the PK parameters, the percent prediction errors were small: −2.02% for Ctrough, −10.92% for C_max,ss_, and −9.82% for AUCτ; all remained well within commonly accepted model performance expectations for such extrapolations in linear PK. Moreover, both observed and simulated test/reference ratios and 90% CIs lay within the 80–125% BE limits, and the simulated steady-state profiles for test and reference overlapped, mirroring the clinical BE (Table 3, Figure 2).

Taken together, these findings indicate that, for an ER formulation exhibiting linear pharmacokinetics, nonparametric superposition of single-dose data can reliably project steady-state exposure and BE.

In the present example, the MIBE outputs were quantitatively concordant with the clinical steady-state study, suggesting that—if applied prospectively within a pre-specified plan and supported by fit-for-purpose checks—the approach could have supported a waiver of the multiple-dose BE requirement, aligning with the broader MIE/MIBE approaches.

### 4.2. Case Study 2: Applicability of MIBE for Long-Acting Injectable Formulations

#### 4.2.1. Objective

To assess whether an MIBE strategy using sparsely sampled concentration–time data can approximate the BE conclusions obtained from complete (rich) PK profiles for an LAI product, thereby reducing operational burden without compromising decision integrity.

#### 4.2.2. Context and Rationale

LAIs often require prolonged sampling windows spanning weeks to months, during which ambulatory visits, late-phase draws, and patient retention pose major execution risks. These trials are typically run as per parallel design across multiple clinical sites, escalating cost and complexity. Sparse sampling, as contemplated within MIBE frameworks as described in the USFDA guidance [33], offers a way to distribute information across subjects by collecting a limited number of strategically timed samples per participant and reconstructing mean profiles at the group level.

In this case example, the approach focuses on preserving information around Cmax while reducing total samples, and then comparing BE metrics derived from sparse data with those from complete profiles to determine whether sparse designs can maintain inferential parity for LAIs.

#### 4.2.3. Methods (MIBE Workflow)

***Tools and software***: The comparative analyses—PK parameter estimation and BE statistics—were performed in Phoenix WinNonlin (v8.6). Sparse profile construction and noncompartmental analyses (NCA) followed the same platform workflow used for the complete profiles to ensure methodological consistency across datasets.

***Study data and design***: Complete plasma PK profiles were available for both test and reference LAI products (n = 15 per arm). NCA yielded Cmax, AUC1–10 day, AUC10–28 day, AUC0–t, and AUC0–∞ for each product, followed by conventional BE calculations.

To create a sparse dataset with the same sample size, time points were reduced while retaining rich sampling around Cmax (Days 3, 5, and 7). For nominal times omitted in the sparse scheme, group-mean concentrations from the complete dataset were used to reconstruct the mean profile at each time point for test and reference, after which the same NCA and BE computations were applied. This allowed a like-for-like comparison of BE outcomes between complete and sparse designs for identical endpoints and population sizes.

***Simulation and analysis***: The central analysis compared test/reference geometric mean ratios with 90% confidence intervals obtained from complete versus sparse datasets across the predefined PK endpoints. Agreement was evaluated using percent error (%PE) on the BE ratios from the sparse dataset relative to the complete-profile benchmark. Visual concordance between the mean observed profiles and the sparse-derived mean profiles was inspected to confirm that the sparse reconstruction retained the essential features of the LAI concentration–time course, particularly the peak region critical for Cmax.

#### 4.2.4. Results and Conclusions

**Figure 3:**
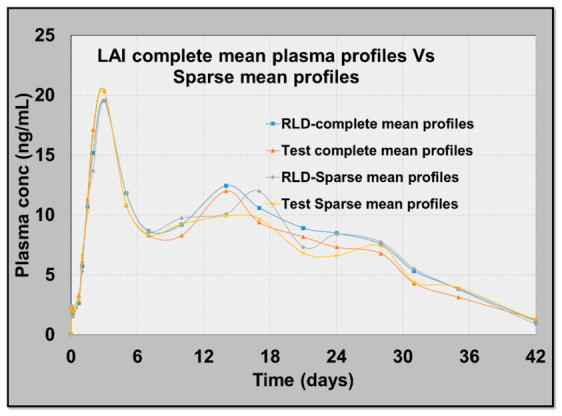
Comparative mean observed plasma profiles vs. sparse mean profiles of both test and reference products.

BE outcomes derived from the sparse dataset closely matched those from the complete profiles across all endpoints. The absolute percent error on the BE ratios remained within approximately ±5%, indicating strong agreement of test and reference comparisons between the two designs (Table 4). The reconstructed mean concentration–time curves for both test and reference were in close visual agreement with the observed mean profiles from the complete dataset, including the peak region, supporting the reliability of the sparse approach for capturing rate- and extent-of-exposure attributes relevant to BE (Figure 3).

This suggests that, with appropriate selection of sampling points—especially ensuring rich sampling near Cmax—a sparse sampling framework can reliably approximate the full PK behavior of LAI formulations. The approach preserved the decision-making signals for both rate and extent metrics while substantially reducing the sampling intensity that often threatens feasibility in LAI trials.

### 4.3. Case Study 3: Applicability of MIBE for Justification of Pivotal Fed BE Study Waiver for High-Risk Products

#### 4.3.1. Objective

To evaluate whether an MIBE approach integrating pilot BE data with an absorption-driven PBBM-based approach can scientifically justify a waiver of the pivotal fed-state BE study for a high-risk immediate-release (IR) formulation developed using an amorphous solid dispersion (ASD) strategy.

#### 4.3.2. Context and Rationale

High-risk IR products—such as ASDs, lipid-based systems, or other enabling technologies—are often sensitive to gastrointestinal conditions, prompting regulatory expectations for both fasting and fed BE studies. In the present case, the RLD contains the API in a crystalline form, whereas the test product uses an ASD to overcome biopharmaceutic and intellectual property constraints.

Although the formulation approach differs, development efforts aimed to ensure comparable in vivo performance across prandial states. Given the recognized food effect for the RLD and the intrinsic biopharmaceutic risk associated with ASDs, a conventional pathway would require a pivotal fed BE study.

This case examines whether integrating observed pilot BE data with a validated PBBM can provide sufficient evidence, within the MIBE framework, to support waiving the pivotal fed study while retaining a pivotal fasting BE assessment, consistent with the manuscript’s broader discussion on context-of-use-driven modeling.

#### 4.3.3. Methods (MIBE Workflow)

***Tools and software***: A PBBM model was developed and applied using GastroPlus^®^ (v9.9.0002) that integrated physicochemical properties, formulation attributes, gastrointestinal physiology, and dissolution behavior. Model fitting, simulation, and comparison with observed data were performed within the same platform.

***Study data and Model development***: Pilot crossover BE studies were conducted under both fasting and fed conditions for the test formulation relative to the RLD. Pilot fasting RLD data were first used to construct the base PBBM, incorporating drug physicochemical parameters (including pH–solubility profile of plain API and the ASD premix, solubility data in biorelevant media, pKa, log P, etc.), permeability, systemic PK inputs, and in vitro dissolution profiles modeled using a Z-factor (Takano model). Physiological parameters appropriate for fasted and fed states were incorporated to mechanistically capture food effects.

***Model validation and application***: The model was validated by comparing predicted vs. observed fed-state PK profiles for the RLD, along with verification of fed/fasted exposure ratios against both pilot observations and literature-reported food-effect data. Once validated, the model was applied to simulate pilot test-product performance under fasted and fed conditions using formulation-specific dissolution inputs. Finally, the same framework was used to simulate pivotal-scale test formulations, enabling prediction of fed-state BE outcomes in the absence of a clinical fed study. All simulations and comparisons reference tables and figures were provided by contributors without modification.

#### 4.3.4. Results and Conclusions

**Figure 4:**
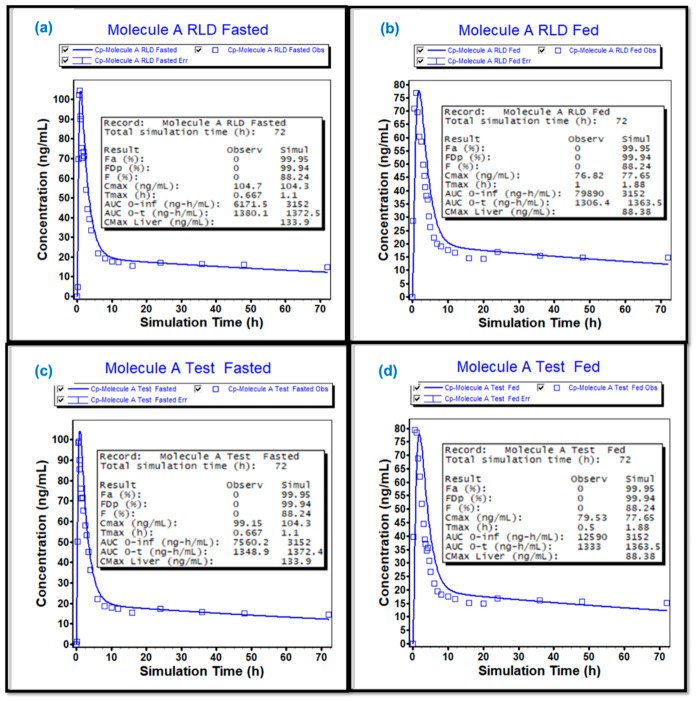
Base model development using (**a**) pilot RLD under fasted state and (**b**) model validation using pilot RLD fed (**c**,**d**) model application for pilot test under fasted and fed states, respectively.

Pilot BE studies demonstrated that the test formulation was bioequivalent to the RLD under both fasting and fed conditions, with test/reference ratios and 90% confidence intervals within 80–125% for C_max_ and AUC (Table 5). The developed PBBM successfully reproduced the observed fasting PK for the RLD and accurately predicted the fed-state profiles, confirming that the model captured the magnitude and direction of the known food effect (Figure 4). Predicted fed/fasted ratios for the RLD were consistent with literature-reported values, providing independent support for model credibility (Table 6).

When applied to the test formulation, the model predicted overlapping fasted and fed profiles with the observed pilot data, and food-effect estimates comparable to both the RLD and published information. Building on this alignment, simulations were extended to the pivotal test formulation, for which a fasting clinical BE study was conducted. The observed pivotal fasting BE outcomes matched the model predictions, establishing internal consistency between simulated and clinical data (Table 5). Importantly, simulated pivotal fed BE ratios remained well within BE limits, indicating a high probability that a clinical fed study would have demonstrated bioequivalence.

Collectively, these findings support a scientific justification for waiving the pivotal fed BE study under the MIBE framework. The convergence of (i) observed pilot fasting and fed BE, (ii) a PBBM validated against RLD food-effect behavior from literature, and (iii) agreement between simulated and observed pivotal fasting outcomes provides coherent evidence that the ASD-based test formulation does not introduce a clinically meaningful food-dependent risk relative to the RLD. This case exemplifies how PBBM-informed MIBE can be leveraged for high-risk products to streamline development while preserving confidence in therapeutic equivalence, consistent with emerging regulatory perspectives discussed earlier in this manuscript.

### 4.4. Case Study 4: Applicability of MIBE for Justification of Failure of PK BE Study Based on Pharmacodynamic Data Support

#### 4.4.1. Objective

To evaluate whether an MIBE approach, integrating steady-state PK projections and pharmacodynamic (PD) considerations can provide a scientifically justified context for a marginal single-dose C_max_ BE failure, thereby supporting regulatory approval for the test product.

#### 4.4.2. Context and Rationale

Regulatory BE assessment for immediate-release oral products requires demonstration of equivalence for both C_max_ and AUC. In this case, a pivotal fasting single-dose BE study met acceptance criteria for AUC, but the 90% confidence interval for C_max_ T/R ratio slightly fell below the lower BE bound (≈80%), resulting in BE failure. However, the molecule under study is characterized by a long elimination half-life and substantial accumulation at steady state—conditions under which differences in peak concentrations observed after a single dose are expected to attenuate with repeated dosing. Supporting this interpretation, public information from the innovator indicates that an earlier formulation change likewise showed reduced single-dose C_max_ without clinical consequence; equivalence was demonstrated at steady state and efficacy was driven primarily by systemic exposure (AUC), not C_max_.

This context motivates use of an MIBE framework to assess the clinical relevance of the observed C_max_ deviation by projecting steady-state behavior and linking PK metrics to PD relevance.

#### 4.4.3. Methods (MIBE Workflow)

***Tools and software***: A nonparametric steady-state simulation approach was applied using Phoenix WinNonlin (v8.6). The platform was used for simulation, noncompartmental analysis, and BE calculations.

***Study data and Model***: Mean observed plasma concentration–time profiles from the pivotal single-dose fasting BE study were used for both test and reference products. Based on published information for the molecule, steady state is reached by Day 15 under the approved dosing regimen. Using the observed single-dose profiles, steady-state concentrations were simulated from Day 0 through Day 15 for each formulation.

***Simulation and analysis***: Simulated profiles at steady state (Day 15) were summarized using NCA to derive C_max_,_ss_ and AUC_τ,ss_. Prior to BE assessment, accumulation ratios from the simulations were compared against literature-reported accumulation values to verify the plausibility of the steady-state projections. Subsequently, test/reference geometric mean ratios with 90% confidence intervals were calculated for the steady-state endpoints and contrasted with the observed single-dose BE results.

#### 4.4.4. Results and Conclusions

**Figure 5:**
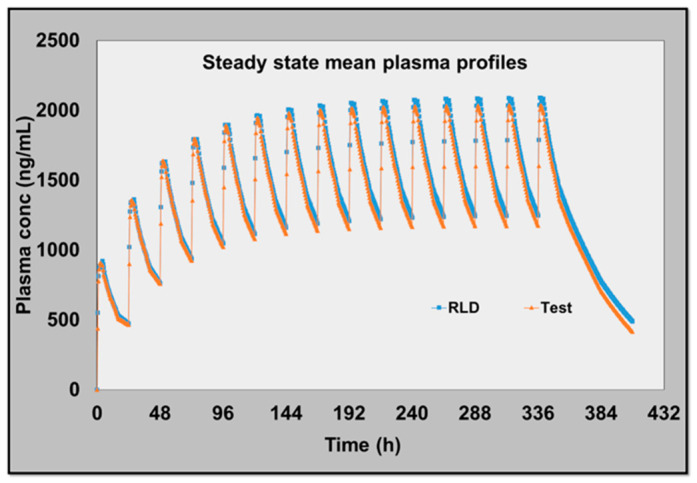
Comparative steady-state simulations for pivotal RLD and test.

Steady-state simulations yielded overlapping concentration–time profiles for the test and reference products, visually indicating convergence in exposure with repeated dosing (Figure 5). The simulated profile-based accumulation ratios for both formulations (~1.8–2.4) were consistent with literature values for this molecule (approximately 2.2–2.5), confirming that the model reasonably captured the drug’s accumulation behavior.

At steady state, the simulated BE analysis demonstrated that both C_max_,ss and AUC_τ,ss_ met conventional BE criteria, with test/reference ratios and 90% confidence intervals entirely within the 80–125% acceptance range (Table 7). In contrast, the single-dose fasting study showed a lower bound of approximately 76% for C_max_, while AUC met BE. The steady-state projections thus illustrate that the apparent single-dose C_max_ discrepancy diminishes upon accumulation, a pattern consistent with the pharmacokinetic properties of long half-life compounds and with the innovator’s own development experience.

Importantly, the pharmacodynamic relevance further supports this conclusion. Available innovator literature indicates that AUC—not C_max_—is the primary driver of efficacy for this molecule, and that modest differences in absorption rate within the observed range do not translate into clinically meaningful differences in safety or therapeutic response. In this context, achievement of BE for AUC at single dose, combined with simulated steady-state equivalence for both C_max_ and AUC, provides a coherent, mechanism-based justification that the marginal single-dose C_max_ miss does not represent a clinically relevant risk.

Overall, this case illustrates how an MIBE framework integrating steady-state PK projections and PD understanding can contextualize borderline BE outcomes. By demonstrating alignment at steady state and reinforcing the exposure–response drivers of clinical effect, the approach supports a science-based interpretation of single-dose C_max_ deviations and exemplifies how model-integrated evidence can augment conventional BE assessments in situations where strict reliance on single-dose PK metrics may be overly conservative.

### 4.5. Case Study 5: MIBE-Based Carryover Correction in Crossover BE Study Due to Short Washout Period

#### 4.5.1. Objective

To demonstrate how an MIBE strategy based on population pharmacokinetic (POP-PK) modeling can correct for significant carryover arising from a short washout period in a 2 × 2 crossover BE study of a long half-life drug, thereby enabling a valid BE assessment when conventional analysis is not feasible.

#### 4.5.2. Context and Rationale

For drugs with long elimination half-life, crossover BE designs are particularly sensitive to washout duration. Traditional approach demands a washout period of at least five half-lives of the drug, which would make the study impractical due to long overall study duration. With a short washout, residual concentrations from Period-1 dosing persist into Period-2, resulting in substantial subject loss due to pre-dose levels exceeding the regulatory threshold (>5% of Period-1 C_max_).

MIBE-based empirically driven POP-PK modeling was used to estimate and remove residual carryover from period-1 while preserving the integrity of the original dataset.

In the present case, the observed crossover BE study data of n = 24 subjects had more than 50% of subjects exhibiting high pre-dose concentrations in Period-2 due to study design with a shorter washout of ~3 half-lives, such that conventional exclusion would have reduced the dataset below the minimum required for BE evaluation.

This scenario makes for a pragmatic setting for applying an MIBE approach to estimate and correct the residual carryover while preserving the integrity of the original dataset.

#### 4.5.3. Methods (MIBE Workflow)

***Tools and software***: A population PK model was developed using the nlmixr2 package in R, and standard BE statistics on corrected data were derived using conventional NCA/BE workflows. Model diagnostics, simulation, and subject-level extrapolation were conducted within the same POP-PK framework in the nlmixr2 package (4.0.0) in R (4.5.1).

***Study data and Design***: The analysis utilized data from a single-dose, two-period, two-sequence crossover BE study (n = 24). More than half of the subjects had Period-2 pre-dose concentrations exceeding 5% of Period-1 C_max_, indicating substantial carryover and precluding conventional BE analysis (Table 8). Rather than excluding these subjects, Period-1 data for all subjects were used to construct a POP-PK model capable of predicting residual concentrations at all Period-2 sampling times.

***Model development and application***: Period-1 concentration–time data were fit to a POP-PK model to estimate typical parameters and individual empirical Bayes estimates (EBEs). Model adequacy was confirmed through standard diagnostics, including goodness-of-fit plots, individual predictions, and visual predictive checks, demonstrating acceptable structural and predictive performance. Using the fitted model, subject-specific residual concentrations attributable to Period-1 dosing were simulated forward to all Period-2 sampling times, including pre-dose. These predicted residuals were then subtracted from the observed Period-2 concentrations on a subject-by-subject, time-matched basis to generate “carryover-corrected” profiles. Small negative values resulting from minor residual prediction error were set to zero prior to non-compartmental analysis, consistent with conventional handling of concentrations below quantification or physiologically implausible negatives.

The corrected Period-2 profiles, together with unaltered Period-1 data, were subjected to standard NCA to compute C_max_ and AUC endpoints, followed by conventional two one-sided *t*-test (TOST) BE analysis.

#### 4.5.4. Results and Conclusions

**Figure 6:**
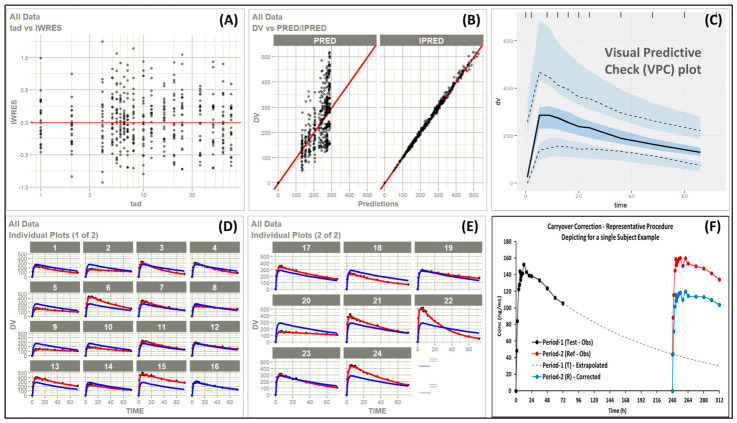
Diagnostic plots from POP-PK model fit—(**A**) individual weighted residuals (IWRES) plotted against time after dose (tad) for all observations. (**B**) Population Predictions (PRED) and Individual Predictions (IPRED) vs observed concentrations for all observed data. (**C**) VPC comparing observed versus simulations PK Profiles—Solid black line: Observed median, Dashed black lines: Observed 5th and 95th percentiles, Shaded regions represent prediction intervals from simulations. (**D**,**E**) Observed (points) and model-predicted (lines) for individual PK profiles—Blue line: Population prediction, Red line: individual prediction. (**F**) Representative individual subject data (Subj-9) depicting the procedure adapted for carryover correction.

In the observed data from the BE study, 13 of 24 subjects exhibited Period-2 pre-dose concentrations above the regulatory threshold, with several subjects exceeding 20% of Period-1 C_max_, clearly demonstrating significant carryover due to the study design with short washout (Table 8). After model-based correction (Figure 6A–D), all subjects’ pre-dose concentrations were reduced to well below the threshold, typically <1% of Period-1 C_max_ (Table 8). Representative individual profiles illustrate that the POP-PK model credibly extrapolated Period-1 disposition and isolated the residual component contributing to Period-2 contamination (Figure 6E).

Importantly, BE analysis performed on the carryover-corrected dataset (n = 24) showed that test and reference formulations met BE criteria for both C_max_ and AUC, with test/reference ratios and 90% confidence intervals fully contained within the 80–125% acceptance range (Table 9).

This example illustrates that model-integrated carryover correction can be an effective strategy to design crossover studies for long half-life drugs with short washout, thereby reducing the burden of a long study.

### 4.6. Case Study 6: MIBE-Based Pivotal Study Waiver for an IR Tablet Formulation Using PBBM and Virtual BE

#### 4.6.1. Objective

To demonstrate how an MIBE strategy combining PBBM, virtual bioequivalence (VBE), and bootstrap-based trial replication can be used to justify a waiver of the pivotal in vivo BE study for an IR tablet formulation when a critical biopharmaceutic attribute (CBA) is clearly identified and controlled.

#### 4.6.2. Context and Rationale

The drug is a BCS Class II compound with dissolution-limited absorption. During early development, API particle size (PSD) emerged as a key formulation variable influencing in vitro dissolution and in vivo exposure. Understanding and controlling such CBAs is central to risk-based biopharmaceutics and aligns with regulatory principles enabling alternative BE approaches when mechanistic understanding is strong.

In this context, a bio-predictive dissolution method and an appropriately validated PBBM can provide a quantitative link between formulation attributes and systemic exposure. This case explores whether such integration, supplemented by VBE and bootstrap analyses, can support a pivotal BE study waiver under an MIBE framework.

#### 4.6.3. Methods (MIBE Workflow)

***Tools and software***: A Semi-mechanistic absorption-driven PBBM was developed and applied using GastroPlus^®^ (v9.9.0002). VBE simulations and population trials were conducted within the same platform, while bootstrap resampling and conventional BE statistics were implemented in R using custom scripts and validated standard packages.

***Study data and Design***: Three pilot test batches (T1, T2, T3) were manufactured to differ only in API PSD (D50 ≈ 1.5, 0.45, and 0.60 μm, respectively), with all other critical material attributes (CMAs), critical formulation variables (CFVs), and process parameters held constant. In vitro dissolution testing in a quality-control (QC) medium (0.1 N HCl + 0.25% SLS, 900 mL, paddle, 50 rpm) demonstrated clear PSD-dependent rank order. A randomized four-way crossover fasting BE study compared each pilot batch to the reference listed drug (RLD). The results showed infra-BE for T1, supra-BE for T2, and BE for T3, confirming API PSD as the governing CBA for in vivo performance.

***Model development, validation and application***: A semi-mechanistic PBBM was constructed using drug physicochemical properties, permeability, disposition parameters, and formulation-specific dissolution inputs incorporated via Z-factor calibration. The model was validated using VBE, demonstrating that simulated populations reproduced both the mean PK profiles and inter-subject variability observed clinically for the RLD and all three pilot batches. This validation effectively established a Level-A-like IVIVC across the PSD design space. Using the validated model, dissolution data from the optimized exhibit batch (EB) manufactured at commercial scale with target PSD were incorporated to simulate pivotal-scale exposure.

***Virtual BE and bootstrap analysis***: A virtual pivotal crossover BE study (n = 36) comparing the Test Exhibit Batch to the RLD was simulated under fasting conditions. To further evaluate robustness, 1000 bootstrap replicate BE trials (each n = 36) were generated from the virtual population data. Each replicate underwent standard BE evaluation to quantify the proportion of trials meeting the 80–125% criteria for C_max_ and AUC.

#### 4.6.4. Results and Conclusions

**Figure 7:**
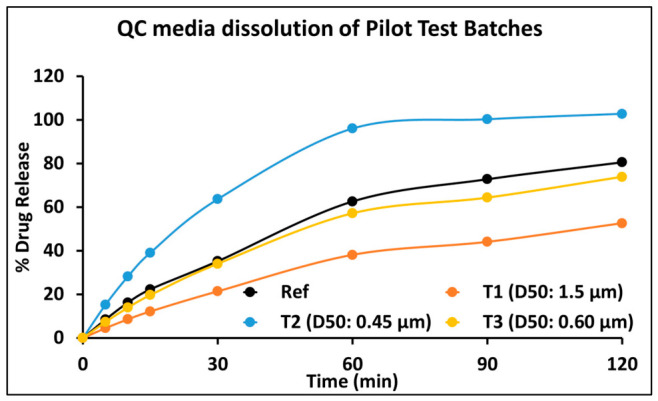
Mean in vitro dissolution of RLD and pilot test batches of Drug B in QC media (n = 12) (0.1N HCl + 0.25% SLS/900 mL/Paddle/50 rpm).

**Figure 8:**
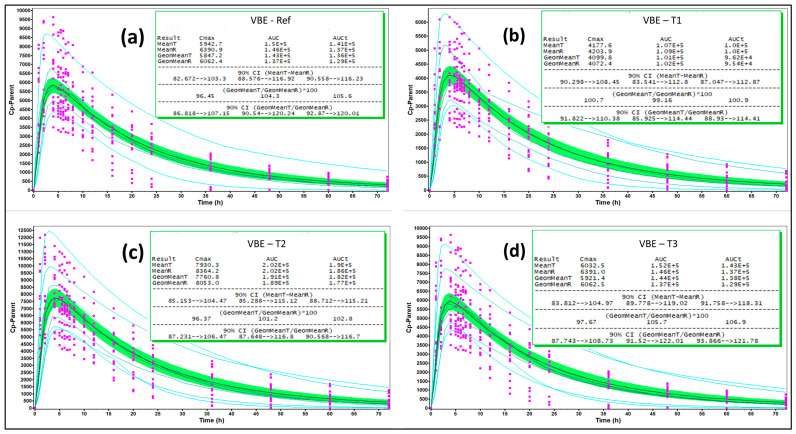
Population simulation-based virtual bioequivalence (VBE) outcome for pilot fasting BE study for (**a**) RLD and test batches—(**b**) T1, (**c**) T2 and (**d**) T3 (n = 16). Note: Pink squares are the observed values; solid line represents mean simulated profile; 90% CI is defined by green band; cyan blue lines represent probability contours.

The pilot BE study (Table 10) established a clear and mechanistic relationship between API PSD, dissolution rate, and systemic exposure. The PBBM, calibrated with QC dissolution data (Figure 7), successfully predicted the observed PK for all pilot batches and the RLD, including population variability, satisfying internal validation criteria (Figure 8).

**Figure 9:**
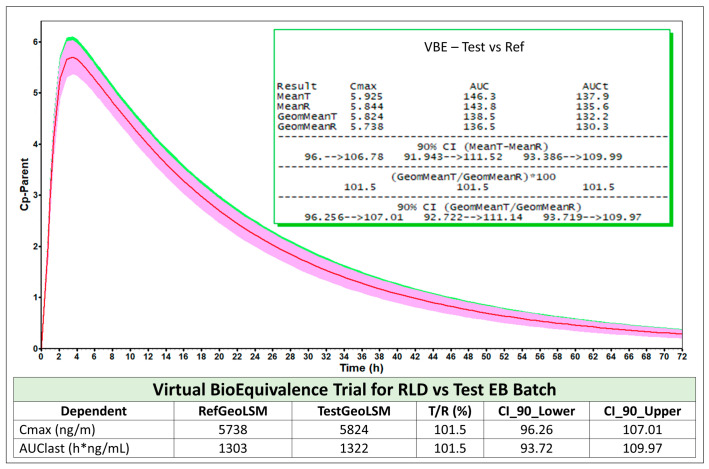
VBE outcome for pivotal test vs. reference formulations in fasted state along with the summary BE statistics from the predicted population (n = 36). This shows that the EB batch was bioequivalent to the RLD formulation under fasted state, based on the developed PBBM-VBE-based IVIVC for the product.

**Figure 10:**
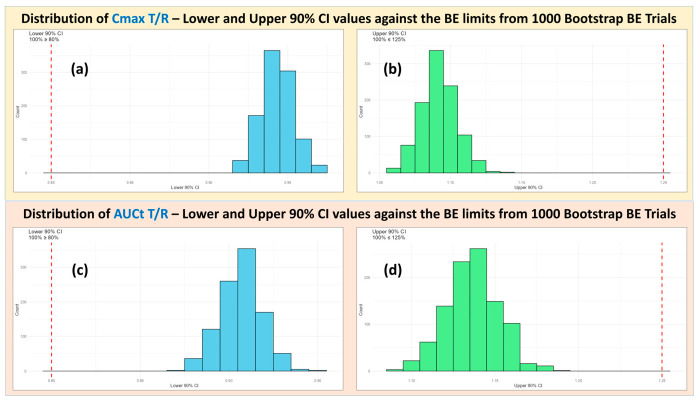
Distribution of the lower and upper 90% CI values of C_max_ (**a**,**b**) and AUC T/R (**c**,**d**) values from 1000 bootstrap BE trials.

For the optimized EB formulation, the virtual pivotal BE simulation demonstrated T/R ratios for C_max_ and AUC comfortably within BE limits (Figure 9). The subsequent bootstrap analysis showed that all (≥95%) of the replicated trials met conventional BE acceptance criteria, providing strong quantitative evidence that a real pivotal study would be highly likely to succeed (Figure 10).

Together, these findings support an MIBE-based waiver of the pivotal in vivo BE study for this IR product. The convergence of (i) a clearly identified and controlled CBA (API PSD), (ii) a bio-predictive dissolution method, (iii) a validated PBBM capable of predicting both mean exposure and variability, and (iv) robust VBE and bootstrap confirmation provides a coherent scientific basis for waiving the clinical study.

This case illustrates how, for IR products with low residual biopharmaceutic risk once CBAs are controlled, model-integrated evidence can rationally replace conventional pivotal BE testing, consistent with emerging regulatory thinking.

## 5. Conclusions

Model-Integrated Bioequivalence (MIBE) is increasingly emerging as a transformative approach in generic drug development, with the potential to reduce reliance on unnecessary clinical bioequivalence studies, improve development efficiency, and minimize subject burden—particularly for complex drug products. A broad spectrum of quantitative modeling tools—including PBPK, PBBM, and POP-PK models—are now available, and appropriate selection should be driven by product characteristics, data availability, and the specific regulatory question of interest. Importantly, the current paradigm is shifting from the historical use of ‘models as supportive tools’ toward ‘reliance on models as critical or pivotal evidence for regulatory decision-making’. Such use necessitates early engagement with regulatory agencies, pre-specified context of use, rigorous model verification and validation, uncertainty characterization, and robust credibility assessments. Collectively, this evolution positions MIBE as a key enabler for efficient, science-driven, and patient-centric generic product development.

## Figures and Tables

**Table 1:** Comparison of PBPK, PBBM and POP-PK models [21,23,24].

Aspect	PBPK	PBBM	POP-PK
Purpose	Mechanistic prediction of systemic PK based on whole body physiology	Mechanistic prediction of systemic PK focusing on biopharmaceutics	Accounts for PK variability and covariate effects using statistical models
Components	Organ physiology (volume, blood flow, physiology, enzyme, transporters)	PBPK components together with drug product, formulation, GI physiology	PK parameter distributions, fixed, random effects, covariates, statistical backend
Type	Highly mechanistic	Mechanistic (biopharmaceutics based)	Empirical
Model inputs	Physiology, in vitro ADME, physicochemical data	Physiology, API, formulation properties (e.g., solubility, dissolution)	Observed plasma concentration–time profiles
Output	PK profiles under different clinical scenarios	PK profiles under different scenarios (w.r.t. formulation)	Parameter estimation, variability explanation
Strengths	Exposure change vs. physiology	Exposure change vs. formulation/API	Understanding of variability, uncertainty explanation
Weakness	Does not account for formulation differences	Extensive formulation characterization, data requirements	Less mechanistic explanation
Treatment of variability	Physiological variability	Physiological + formulation variability	Random effects + covariates
Simulating formulation changes	Low	High	Low
Regulatory utility	Food effects, special population predictions, DDIs	Virtual BE, IVIVC/R, biowaivers	Model-based BE, Virtual BE, PK bridging
Regulatory acceptance	High (FDA and EMA)	Growing rapidly; FDA MIBE submissions are commonly PBBM	Very high for BE, PK bridging, shortening BE duration

**Table 2:** Overview of the case studies discussed in the manuscript summarizing the conventional BE approach and the corresponding MIBE-based alternative for each development scenario or question.

Case Study No.	Problem/Opportunity Statement	Conventional Approach	Alternative Approach (MIBE Based)	Advantages of Alternative Approach (Cost and Time)
1	MIBE for waiver of multiple-dose BE study for extended-release formulations	As per EMA guidance for MR products, if there is accumulation, need to perform single dose and steady-state (multiple-dose) BE studies	Demonstrate MIBE to waive steady state BE for ER formulation.	Waiver of multiple-dose BE studies leads to significant cost and time savings and can ensure early approval of product to reach patient needs.
2	MIBE for long-acting injectables	Generally rich sampling is required based on literature profiles, T_max_ and half-life data and need to prove BE with complete profile data	Applicability of MIBE using sparse PK data vs. full profiles.	Significant reduction in cost, and time savings and can ensure early approval of product to reach patient needs.
3	MIBE for pivotal fed BE study waiver for high-risk IR oral product	As per ICH M13 A both fasting and fed BE studies are required	Support fed-state BE waiver for high-risk drug.	Fasting or fed BE study can be waived based on label recommendation leads to significant cost and time savings and can ensure early approval of product to reach patient needs.
4	MIBE to justify failed BE (PD-based)	Need to perform BE study, if product fails redevelopment to get the new BE study success	Support C_max_ failure justification using PD evidence.	New development cost can be waived and leads to significant cost and time savings and can ensure early approval of product to reach patient needs.
5	Carryover effect (high pre-dose concentrations) due to short washout for a long half-life drug	Perform BE study with >5 half-lives of washout to avoid carryover effect If the drug has a half-life of 48 h, ≥10 days of washout is required, which adds significant cost and duration for the study and increases chances of drop-outs	Perform a study with short washout (1–2 half-lives). Extrapolate individual subject Period-1 residual concentrations to Period-2. Remove carry over based on the extrapolated data and demonstrate BE.	Significant cost and time savings as the BE study duration could be significantly reduced.
6	Waiver of pivotal BE study for IR tablet of BCS class II drug with pilot study data, CBA and bio-predictive method identified	Perform a full pivotal BE study with required number of subjects and study duration	IR product with Pilot Study data, CBA and bio-predictive dissolution method identified. Hence, can be categorized as low biopharmaceutics risk product. Justify pivotal study waiver using Pilot—large scale similarity of product. PBBM—VBE-based modeling to demonstrate that test exhibit batch is BE to RLD. Bootstrap BE-based population simulations to demonstrate very low risk.	Significant cost and time savings as the pivotal BE study could be waived, which would be required as per conventional approach.

**Table 3:** Observed BE summary statistics from single-dose fasted-state BE study, and observed vs. predicted multiple-dose study.

**Single-Dose Observed Bioequivalence (n = 27)**			
**PK Parameter**	**Reference** **Geometric Mean**	**Test** **Geometric Mean**	**Test/Reference Ratios (CI Limits)**
C_max_ (ng/mL)	92.14	94.59	102.66 (97.05–108.60)
AUC_0–24_ (ng·h/mL)	1437.60	1442.83	100.36 (92.50–108.89)
AUC_0–t_ (ng·h/mL)	1685.35	1676.23	99.46 (90.72–109.04)
AUC_0–inf_ (ng·h/mL)	1698.45	1690.00	99.50 (90.77–109.07)
Accumulation ratios	**84.64**	**85.37**	
**Multiple dose at 6th dose observed vs. simulated bioequivalence (n = 27)**			
**PK parameter**	**Test Multiple-dose** **Observed BE ratios** **(CI limits) n = 27**	**Test Multiple-dose** **Predicted BE ratios** **(CI limits) n = 27**	**% Prediction error**
C_tss_ (ng/mL)	94.98 (86.39–104.43)	96.90 (81.53–115.17)	**−2.02**
C_max_ (ng/mL)	89.16 (84.73–93.76)	98.90 (92.88–105.32)	**−10.92**
AUC_0-tau_ (ng·h/mL)	90.59 (85.86–95.60)	99.49 (90.77–109.04)	**−9.82**

**Table 4:** Long-acting injectable formulations complete profiles observed BE data vs. sparse samples BE data.

Bioequivalence—Test/Reference Ratios (CI Limits)			
PK Parameter	Complete Profiles BE Outcome	Sparse Profiles BE Outcome After Simulations	% PE
C_max_ (ng/mL)	106.73 (94.88–120.07)	105.30 (93.56–118.51)	1.35
AUC_1–10 day_ (ng·h/mL)	99.25 (90.18–109.23)	100.28 (91.15–110.32)	−1.04
AUC_10–28 day_ (ng·h/mL)	88.83 (80.68–97.81)	88.43 (83.86–93.26)	0.45
AUC_0–t_ (ng·h/mL)	91.11 (83.24–99.73)	93.33 (88.31–98.63)	−2.44
AUC_0–Inf_ (ng·h/mL)	92.95 (85.93–100.55)	95.12 (90.44–100.04)	−2.33

**Table 5:** High-risk molecule-A, pilot fasting, fed and pivotal fasting observed bioequivalence observed data vs. simulated outcome.

**Fasting Bioequivalence—Test/Reference Ratios (CI Limits)**			
**PK Parameter**	**Pilot Observed n = 14**	**Simulated Before Pivotal** **at n = 28**	**Pivotal** **Observed n = 36**
C_max_ (ng/mL)	98.52 (82.65–117.43)	98.50 (87.88–110.43)	99.8 (89.95–110.73)
AUC_0–t_ (ng·h/mL)	96.32 (79.41–116.83)	96.30 (84.95–109.20)	98.2 (87.60–110.08)
**Fed bioequivalence—Test/Reference ratios (CI limits)**			
**PK parameter**	**Pilot Observed n = 15**	**Simulated before pivotal** **at n = 36**	**Pivotal**
C_max_ (ng/mL)	100.6 (82.35–122.85)	100.5 (88.24–114.65)	**Fed waiver**
AUC_0–t_ (ng·h/mL)	101.6 (87.46–118.11)	101.0 (92.12–112.14)	

**Table 6:** RLD and test product observed vs. predicted food effect and comparison against literature-reported data.

**PK Parameter**	**Pilot RLD** **Fed/Fasted State Observed Ratios**	**Pilot Test** **Fed/Fasted State Observed Ratios**	**Literature-Reported Data**
C_max_	69.44	70.90	~60
AUC_0–t_	85.74	90.48	88
**PK parameter**	**Pilot RLD** **Fed/Fasted state Predicted ratios**	**Pilot test** **Fed/Fasted state Predicted ratios**	**-**
C_max_	74.5	74.6	-
AUC_0–t_	99.3	99.4	-

**Table 7:** Test product fasting bioequivalence single dose vs. multiple dose.

PK Parameter	Test Single-Dose Observed BE Ratios vs. RLD (CI Limits) n = 12	Test Multiple-Dose Predicted BE Ratios vs. RLD at 15^th^ Dose (CI Limits) n = 12
C_max_ (ng/mL)	88.52 (76.12–102.94)	91.84 (84.24–100.12)
AUC_0–t_ (ng·h/mL)	98.38 (93.04–104.02)	94.58 (83.39–107.27)

**Table 8:** Subject-wise observed and MIBE-corrected pre-dose concentrations from the crossover BE study.

Subject No.	Period-1 C_max_ (ng/mL)	Pre-Dose Conc (ng/mL)		% Pre-Dose (of P-1 C_max_)	
		Observed	Corrected *	Observed	Corrected *
S-1	233.89	5.40	0.09	2.31%	0.04%
S-2	168.57	38.92	0.67	23.09%^$^	0.40%
S-3	330.48	0.84	−0.02	0.26%	−0.01%
S-4	307.40	1.42	−0.14	0.46%	−0.05%
S-5	174.23	14.73	0.18	8.45%^$^	0.10%
S-6	434.86	18.54	3.37	4.26%	0.78%
S-7	343.19	18.98	5.65	5.53%^$^	1.65%
S-8	228.64	56.20	2.67	24.58%^$^	1.17%
S-9	152.18	44.12	−0.23	29.00%^$^	−0.15%
S-10	199.78	24.65	3.03	12.34%^$^	1.52%
S-11	336.64	28.37	10.07	8.43%^$^	2.99%
S-12	292.83	45.53	10.36	15.55%^$^	3.54%
S-13	396.07	62.69	12.29	15.83%^$^	3.10%
S-14	242.65	19.64	−0.09	8.09%^$^	−0.04%
S-15	502.89	81.71	8.63	16.25%^$^	1.72%
S-16	303.79	12.77	−0.28	4.20%	−0.09%
S-17	357.00	15.30	0.63	4.29%	0.18%
S-18	243.43	3.45	0.00	1.42%	0.00%
S-19	295.54	57.19	6.86	19.35%^$^	2.32%
S-20	164.22	40.85	−0.32	24.88%^$^	−0.20%
S-21	419.46	5.71	−0.48	1.36%	−0.11%
S-22	517.07	0.33	0.13	0.06%	0.03%
S-23	315.29	13.51	5.76	4.28%	1.83%
S-24	446.67	6.69	−0.29	1.50%	−0.06%

* The small negative values are due to minor residual error in the model predictions of Period-1 data, ^$^ Subjects with pre-dose concentrations higher than 5% in period 2.

**Table 9:** Summary statistics from BE analysis of the corrected data of test vs. reference (n = 24).

Dependent	Test_GM	Ref_GM	% T/R	% CI_90_Lower	% CI_90_Upper
C_max_ (ng/mL)	288.42	306.41	94.13	88.40	100.22
AUC0–72 h (ng·h/mL)	13,936.79	14,987.84	92.99	89.14	97.01

**Table 10:** Summary BE statistics from the fasting pilot BE study of T1, T2 and T3 batches against RLD (n = 16).

**T1 vs. R**					
**Dependent**	**RefGeoLSM**	**TestGeoLSM**	**T/R (%)**	**CI_90_Lower**	**CI_90_Upper**
C_max_ (ng/mL)	5951.20	4005.20	67.30	61.06	74.18
AUC_last_ (h·ng/mL)	129,370.02	95,955.22	74.17	68.42	80.40
AUC_inf_ (h·ng/mL)	137,094.26	102,755.90	74.95	68.88	81.56
**T2 vs. R**					
**Dependent**	**RefGeoLSM**	**TestGeoLSM**	**T/R (%)**	**CI_90_Lower**	**CI_90_Upper**
C_max_ (ng/mL)	5951.35	7896.89	132.69	120.52	146.09
AUC_last_ (h·ng/mL)	129,373.28	177,951.66	137.55	126.98	149.00
AUC_inf_ (h·ng/mL)	137,097.69	190,301.89	138.81	127.64	150.95
**T3 vs. R**					
**Dependent**	**RefGeoLSM**	**TestGeoLSM**	**T/R (%)**	**CI_90_Lower**	**CI_90_Upper**
C_max_ (ng/mL)	5951.25	5309.73	89.22	80.96	98.33
AUC_last_ (h·ng/mL)	129,371.11	123,319.06	95.32	87.96	103.30
AUC_inf_ (h·ng/mL)	137,095.40	131,968.62	96.26	88.49	104.72

LSM: Lease square mean.

## Data Availability

Data availability statement is not applicable for this review article as no new data was generated during this publication.

## Abbreviations

The following abbreviations are used in this manuscript:

ANDA	Abbreviated New Drug Application
API	Active Pharmaceutical Ingredient
ASD	Amorphous Solid Dispersion
AUC	Area Under the Plasma Concentration–Time Curve
AUC_0–∞_	Area Under the Curve from Time Zero to Infinity
AUC_τ_	Area Under the Curve over a Dosing Interval
BA	Bioavailability
BE	Bioequivalence
BCS	Biopharmaceutics Classification System
CBA	Critical Biopharmaceutic Attribute
CFR	Code of Federal Regulations
CI	Confidence Interval
CL	Clearance
CMA	Critical Material Attribute
C_max_	Maximum Plasma Concentration
C_max,ss_	Maximum Plasma Concentration at Steady State
CPP	Critical Process Parameter
CRCG	Center for Research on Complex Generics
DDI	Drug–Drug Interaction
EMA	European Medicines Agency
ER	Extended Release
FDA	Food and Drug Administration
f_2_	Similarity Factor for Dissolution Profiles
GI	Gastrointestinal
GM	Geometric Mean
ICH	International Council for Harmonisation
IND	Investigational New Drug
IR	Immediate Release
IVIVC	In Vitro–In Vivo Correlation
IVIVR	In Vitro–In Vivo Relationship
LAI	Long-Acting Injectable
M&S	Modeling and Simulation
MIDD	Model-Informed Drug Development
MIE	Model-Integrated Evidence
MIBE	Model-Integrated Bioequivalence
MR	Modified Release
NDA	New Drug Application
NCA	Non-Compartmental Analysis
NLME	Nonlinear Mixed-Effects (Modeling)
NTI	Narrow Therapeutic Index
PBPK	Physiologically Based Pharmacokinetic (Modeling)
PBBM	Physiologically Based Biopharmaceutics Modeling
PD	Pharmacodynamic(s)
PK	Pharmacokinetic(s)
Pop-PK	Population Pharmacokinetic (Modeling)
QC	Quality Control
QCP	Quantitative Clinical Pharmacology
QMM	Quantitative Modeling Methods
RLD	Reference Listed Drug
SUPAC	Scale-Up and Post-Approval Changes
T/R	Test-to-Reference Ratio
VBE	Virtual Bioequivalence
VPC	Visual Predictive Check
